# Supplementary material for: Comparative Genomics Reveals Species-Specific Genes and Symbiotic Adaptations in Tricholoma matsutake
Source: J Fungi (Basel). 2024 Oct 29;10(11):746. doi: 10.3390/jof10110746 (PMC11595502; doi:10.3390/jof10110746)
Supplement: Supplementary file 1 [file jof-10-00746-s001.zip › jof-3213415-supplementary.pdf]

**Supplementary Tables and Figures**

# Comparative Genomics Reveals Species-specific Genes and Symbiotic Adaptations in *Tricholoma matsutake*

**Jea Hyeoung Kim [1,†], Eun-Kyung Bae [2,†], Yoeguang Hue [1], Byungheon Choi [3], Min-Jeong Kang [2], Eung-Jun Park [2,*] and Ki-Tae Kim [1,4,*]**

[1]Department of Plant Medicine, Sunchon National University, Suncheon 57922, Korea

[2]Forest Microbiology and Application Division, Forest Bioresources Department, National Institute of Forest Science, Suwon 16631, Republic of Korea

[3]Department of Multimedia Engineering, Sunchon National University, Suncheon 57922, Korea

[4]Department of Agricultural Life Science, Sunchon National University, Suncheon 57922, Korea

[*]Correspondence: Eung-Jun Park; Phone) +82-31-290-1171; E-mail) pahkej@korea.kr; Ki-Tae Kim; Phone) +82-61-750-5193; E-mail) kitaekim@scnu.ac.kr

[†]These authors contributed equally to this work.

**Supplementary Table 1. List of *T. matsutake* and *T. bakamatsutake* strains used for PCR amplification.**

| Species | NIFoS[1)] Strain No. | Location | Year of isolation | Host plant | Origin of strain | Genomic DNA | Growth medium | Reference |
|---|---|---|---|---|---|---|---|---|
| *Tricholoma matsutake* | NIFoS 434 | Samcheok, Gangwon-do, South Korea | 1996 | *Pinus densiflora* | Bacidiocarp tissue | Mycelia | Potato Dextrose Agar | Jeon and Ka. 2001 |
| | NIFoS 561 | Pohang, Gyeongsangbuk-do, South Korea | 2001 | *Pinus densiflora* | Bacidiocarp tissue | Mycelia | Potato Dextrose Agar | |
| | NIFoS 2001 | Yeongwol, Gangwon-do, South Korea | 2012 | *Pinus densiflora* | Bacidiocarp tissue | Mycelia | Potato Dextrose Agar | |
| *Tricholoma bakamatsutake* | NIFoS 3833 | Hongcheon, Gangwon-do, South Korea | 2016 | *Quercus mongolica* | Bacidiocarp tissue | Mycelia | Potato Dextrose Agar | Unpublished |
| | NIFoS 4757 | Hongcheon, Gangwon-do, South Korea | 2018 | *Quercus mongolica* | Spore | Mycelia | Potato Dextrose Agar | |
| | NIFoS 4982 | Hongcheon, Gangwon-do, South Korea | 2019 | *Quercus mongolica* | Bacidiocarp tissue | Mycelia | Potato Dextrose Agar | |

1) NIFoS: National Institute of Forest Science

## Reference

Jeon, S.-M.; Ka. K.-H. Korean *Tricholoma matsutake* strains that promote mycorrhization and growth of *pinus densiflora* seedling. *Kor. J. Mycol.* **2001**. 44, 155-165. http://dx.doi.org/10.4489/KJM.2016.44.3.155

**Supplementary Table 2. List of PCR primers used in this study**

| Primer name | Sequence (5' → 3') | Tm (℃) | GC (%) | Size (bp) |
|---|---|---|---|---|
| Tbak-g4359/g4654-F1 | GTGAAAAGCCACCGTGACTTA | 59.7 | 48 | 21 |
| Tbak-g4359/g4654-R1 | GTAATGGCGACGAGGGTTTA | 59.3 | 50 | 20 |
| ITS 1 | TCCGTAGGTGAACCTGCGG | 65.6 | 63 | 19 |
| ITS 4 | TCCTCCGCTTATTGATATGC | 55.8 | 45 | 20 |

*Tricholoma bakamatsutake* is abbreviated as Tbak.

ITS primer pairs are universal fungal primers used as internal controls in this study.

```
g4359_CP114861.1_2337142_2337820_      AATTCAAGTAGGGGCTCCCTGTCCCCAACGAGGGGAATGACAATGTCCGGGAAAGTGAAG   60
g4654_CP114861.1_7857846_7858524_      AATTCAAGTAGGGGCTCCCTGTCCCCAACGAGGGGAATGACAATGTCCGGGAAAGTGAAG   60
                                       ************************************************************

g4359_CP114861.1_2337142_2337820_      ATGGTGTTGGAGAACGTTTGTCCTCGCCATCTGCGTCCATTGAAATCAAGGAACTAATTC   120
g4654_CP114861.1_7857846_7858524_      ATGGTGTTGGAGAACGTTTGTCCTCGCCATCTGCGTCCATTGAAATCAAGGAACTAATTC   120
                                       ************************************************************

g4359_CP114861.1_2337142_2337820_      CTGATGGATATGACATATGAACTTCAGCGCTGGAACTAGGCTGAGGCACTTGGGAAGTTG   180
g4654_CP114861.1_7857846_7858524_      CTGATGGATATGACATATGAACTTCAGCGCTGGAACTAGGCTGAGGCACTTGGGAAGTTG   180
                                       ************************************************************

g4359_CP114861.1_2337142_2337820_      GTGAATGGAAGAAAGATCCGACATACGAATTACTATTCTCCTGGTTGCTTGAAGCGAAAT   240
g4654_CP114861.1_7857846_7858524_      GTGAATGGAAGAAAGATCCGACATACGAATTACTATTCTCCTGGTTGCTTGAAGCGAAAT   240
                                       ************************************************************

g4359_CP114861.1_2337142_2337820_      TTGGATTCGCATGGTTCCGAGACAACGTGGACAAGCCGACTTTAAATGATAGTGATGACT   300
g4654_CP114861.1_7857846_7858524_      TTGGATTCGCATGGTTCCGAGACAACGTGGACAAGCCGACTTTAAATGATAGTGATGACT   300
                                       ************************************************************

g4359_CP114861.1_2337142_2337820_      GAAGCGGGCTCTTTCTAGGTTGCGTGAACGCAGGCGCGTCGTTCTGCGTGGTGGCAAGGG   360
g4654_CP114861.1_7857846_7858524_      GAAGCGGGCTCTTTCTAGGTTGCGTGAACGCAGGCGCGTCGTTCTGCGTGGTGGCAAGGG   360
                                       ************************************************************

g4359_CP114861.1_2337142_2337820_      GTGAGGGCAATGGATATGCTTTCGCTCCACGTTGATGTGCATGGTGGAAATAGAGATTCT   420
g4654_CP114861.1_7857846_7858524_      GTGAGGGCAATGGATATGCTTTCGCTCCACGTTGATGTGCATGGTGGAAATAGAGATTCT   420
                                       ************************************************************

g4359_CP114861.1_2337142_2337820_      CTACTTCGTTCAGATTTTGTTTTTGCTCGAAAGAGACAGGATGAGCATTTCATGAAGGGT   480
g4654_CP114861.1_7857846_7858524_      CTACTTCGTTCAGATTTTGTTTTTGCTCGAAAGAGACAGGATGAGCATTTCATGAAGGGT   480
                                       ************************************************************

g4359_CP114861.1_2337142_2337820_      CACAAGGAGAACGACATACCCAGCCATGGTCTACCTTCAACCGAGCATATTGGAGACGCA   540
g4654_CP114861.1_7857846_7858524_      CACAAGGAGAACGACATACCCAGCCATGGTCTACCTTCAACCGAGCATATTGGAGACGCA   540
                                       ************************************************************

g4359_CP114861.1_2337142_2337820_      TTTGGAGTTGTCGTGAAAGCTACAGATTAAGGGTGTCTAGTATCATTCATTCAGATAAGA   600
g4654_CP114861.1_7857846_7858524_      TTTGGAGTTGTCGTGAAAGCTACAGATTAAGGGTGTCTAGTATCATTCATTCAGATAAGA   600
                                       ************************************************************
```

```
g4359_CP114861.1_2337142_2337820_      CGGACGTACGTGATTAACCTTCGTCCTTTCGATGGTCTGCTTCCTCCGCCTTTCGGTCTC  660
g4654_CP114861.1_7857846_7858524_      CGGACGTACGTGATTAACCTTCGTCCTTTCGATGGTCTGCTTCCTCCGCCTTTCGGTCTC  660
                                       ************************************************************

g4359_CP114861.1_2337142_2337820_      GTTCGTTTGATTGTTCGCAGCCGACGTCGGGCTGATCATCCCGGTTCAATGAAGATGTAT  720
g4654_CP114861.1_7857846_7858524_      GTTCGTTTGATTGTTCGCAGCCGACGTCGGGCTGATCATCCCGGTTCAATGAAGATGTAT  720
                                       ************************************************************

g4359_CP114861.1_2337142_2337820_      CTTACAGATAGAGCGAACAAAGTATTCGAAGGGACGCAGAGGAGATGCCAGGCCAGTGGC  780
g4654_CP114861.1_7857846_7858524_      CTTACAGATAGAGCGAACAAAGTATTCGAAGGGACGCAGAGGAGATGCCAGGCCAGTGGC  780
                                       ************************************************************

g4359_CP114861.1_2337142_2337820_      GGCTGTAGCGTTGCACAGCGCCAATGGCGTTTGAAAAACTCGAGGCACATGTAGTGAAGT  840
g4654_CP114861.1_7857846_7858524_      GGCTGTAGCGTTGCACAGCGCCAATGGCGTTTGAAAAACTCGAGGCACGTGTAGTGAAGT  840
                                       ************************************************ ***********

                                                             Tbak-g4359/g4654-F1
g4359_CP114861.1_2337142_2337820_      CATGTGAGTTCTCAAATTCATCACGTGAAAAGCCACCGTGACTTACTCAGCGCGCTGCGC  900
g4654_CP114861.1_7857846_7858524_      CATGTGAGTTCTCAAATTCATCACGTGAAAAGCCACCGTGACTTACTCAGCGCGCTGCGC  900
                                       ************************************************************

g4359_CP114861.1_2337142_2337820_      CGGCGTTCCGGGTTGTAATGTTTTCTTGTACTGTTAGTCTTTATTTCTGCTCAACTCCTA  960
g4654_CP114861.1_7857846_7858524_      CGGCGTTCCGGGTTGTAATGTTTTCTTGTACTGTTAGTCTTTATTTCTGCTCAACTCCTA  960
                                       ************************************************************

g4359_CP114861.1_2337142_2337820_      TTCAGAGAGGGTTCAGATACACGTGTCATTGAAGACGGCCATGCCGCTTTCTCCTCCCCT  1020
g4654_CP114861.1_7857846_7858524_      TTCAGAGAGGGTTCAGATACACGTGTCATTGAAGACGGCCATGCCGCTTTCTCCTCCCCT  1020
                                       ************************************************************

g4359_CP114861.1_2337142_2337820_      CAATTTCCGATCGTGGTTATCTGAGAATGAGCATCTGCTTCAGCCACCCGTGAACAATTT  1080
g4654_CP114861.1_7857846_7858524_      CAATTTCCGATCGTGGTTATCTGAGAATGAGCATCTGCTTCAGCCACCCGTGAACAATTT  1080
                                       ************************************************************

g4359_CP114861.1_2337142_2337820_      TTGCATGTACAAAGGCGGTGACTTCATCGTGATGGCGGTGGGAGGGCCCAATGAAAGGAA  1140
g4654_CP114861.1_7857846_7858524_      TTGCATGTACAAAGGCGGTGACTTCATCGTGATGGCGGTGGGAGGGCCCAATGAAAGGAA  1140
                                       ************************************************************

g4359_CP114861.1_2337142_2337820_      GGATTATCATGTGAATGAAACGGAGGTCAGGCTAGTTTGCCTGACCCACCTGTGGGACCA  1200
g4654_CP114861.1_7857846_7858524_      GGATTATCATGTGAATGAAACGGAGGTCAGGCTAGTTTGCCTGACCCACCTGTGGGACCA  1200
                                       ************************************************************
```

```
g4359_CP114861.1_2337142_2337820_      CTAATGCTTTGCGGTAGGAGTGGTTTTATCAGCACAAGGGCGGGATGTTGTTACGGACTG   1260
g4654_CP114861.1_7857846_7858524_      CTAATGCTTTGCGGTAGGAGTGGTTTTATCAGCACAAGGGCGGGATGTTGTTACGGACTG   1260
                                       ************************************************************

g4359_CP114861.1_2337142_2337820_      TAGACGGCGATAAATTCAGAGACATCAGAATCGAAGAAGGGGATATGTTTTTATTACCTG   1320
g4654_CP114861.1_7857846_7858524_      TAGACGGCGATAAATTCAGAGACATCAGAATCGAAGAAGGGGATATGTTTTTATTACCTG   1320
                                       ************************************************************

g4359_CP114861.1_2337142_2337820_      GTGAGGAGCGATATTACCTCTGTCCTGTTTTGTCCAACGCTGCTGCAGCCAACACACCTC   1380
g4654_CP114861.1_7857846_7858524_      GTGAGGAGCGATATTACCTCTGTCCTGTTTTGTCCAACGCTGCTGCAGCCAACACACCTC   1380
                                       ************************************************************

g4359_CP114861.1_2337142_2337820_      ATAATCCGGTGCGATACGCGAACACGATTGGACTGGTGGTTGAACGTGTAAGGCCACCAC   1440
g4654_CP114861.1_7857846_7858524_      ATAATCCGGTGCGATACGCGAACACGATTGGACTGGTGGTTGAACGTGTAAGACCACCAC   1440
                                       **************************************************** *******

g4359_CP114861.1_2337142_2337820_      AGTCAATTGGCAAGTCTTTTCTCTTCTTTTCCAAACTCTTCCCTGCTCAATTGTGCTATT   1500
g4654_CP114861.1_7857846_7858524_      AGTCAATTGGCAAGTCTTTTCTCTTCTTTTCCAAACTCTTCCTTGCTCAATTGTGCTATT   1500
                                       ****************************************** *****************

g4359_CP114861.1_2337142_2337820_      CAGACCGTCTTCGTTGGTATTGTCCTTCATCCACTCACGCGGAGCCTACGATCATCTACG   1560
g4654_CP114861.1_7857846_7858524_      CAGACCGTCTTCGTTGGTATTGTCCTTCATCCACTCACGCGGAGCCTACGATCATCTACG   1560
                                       ************************************************************

g4359_CP114861.1_2337142_2337820_      AAGAGTCGTTTCACGTCACAGATTTGGGCACTCAGCTCAAACCTGTTATCGAACGTTGGA   1620
g4654_CP114861.1_7857846_7858524_      AAGAGTCGTTTCACGTCACAGATTTGGGCACTCAGCTCAAACCTGTTATCGAACGTTGGA   1620
                                       ************************************************************

g4359_CP114861.1_2337142_2337820_      TGACGCAGGAGGATCTCAGGAAATGTAAACTTTGCGGCACGGCGGCCGACGCTAAGTAAC   1680
g4654_CP114861.1_7857846_7858524_      TGACGCAGGAGGATCTCAGGAAATGTAAACTTTGCGGCACGGCGGCCGACGCTAAGTAAC   1680
                                       ************************************************************

g4359_CP114861.1_2337142_2337820_      AGAATCGCGCTGTATTTAAGTATCTGTGATTTATCAGTTATCTATTGCACATCCTGTCGA   1740
g4654_CP114861.1_7857846_7858524_      AGAATCGCGCTGTATTTAAGTATCTGTGATTTATCAGTTATCTATTGCACATCCTGTCGA   1740
                                       ************************************************************

g4359_CP114861.1_2337142_2337820_      TGATCACATAGAACACACTCATGACATTGAAAGGCAGTGCTCCTTAATGCACGCTGTCGC   1800
g4654_CP114861.1_7857846_7858524_      TGATCACATAGAACACACTCATGACATTGAAAGGCAGTGCTCCTTAATGCACGCTGTCGC   1800
                                       ************************************************************
```

```
g4359_CP114861.1_2337142_2337820_    AAGTCGTATGGGGCTACAGATACTCCGATACCACGTTTACTATTTAGTGCGATACATTAG   1860
g4654_CP114861.1_7857846_7858524_    AAGTCGTATGGGGCTACAGATACTCCGATACCACGTTTACTATTTAGTGCGATACATTAG   1860
                                     ************************************************************

g4359_CP114861.1_2337142_2337820_    CAAGCAGCAGGCAAGTACATATATGCAATCTGCCACGTATCATTGGACCTCGGCGCTCAG   1920
g4654_CP114861.1_7857846_7858524_    CAAGCAGCAGGCAAGTACATATATGCAATCTGCCACGTATCATTGGACCTCGGCGCTCAG   1920
                                     ************************************************************

g4359_CP114861.1_2337142_2337820_    CTTCCATGCTCTAAGATAAGAAAAAGAGAAGATTGACGACAGCACGATTCTCGGTTCCAC   1980
g4654_CP114861.1_7857846_7858524_    CTTCCATGCTCTAAGATAAGAAAAAGAGAAGATTGACGACAGCACGATTCTCGGTTCCAC   1980
                                     ************************************************************

g4359_CP114861.1_2337142_2337820_    CGTGAACAATGCGGCCGCTGTGCATTTGGCAATTCCAGCGGATGATCTGGATCAGACCAT   2040
g4654_CP114861.1_7857846_7858524_    CGTGAACAATGCGGCCGCTGTGCATTTGGCAATTCCAGCGGATGATCTGGATCAGACCAT   2040
                                     ************************************************************

g4359_CP114861.1_2337142_2337820_    CCTTCGGTGTCACTATATACTTCCTTCCTTCTTCGATGGTTCCATTCACATGTCAAGTCA   2100
g4654_CP114861.1_7857846_7858524_    CCTTCGGTGTCACTATATACTTCCTTCCTTCTTCGATGGTTCCATTCACATGTCAAGTCA   2100
                                     ************************************************************

g4359_CP114861.1_2337142_2337820_    ATGTCGCATCGCCATCATGCGACGCACGATCTTAGTTGTAACTGGCTCAGATAGGCGCCA   2160
g4654_CP114861.1_7857846_7858524_    ATGTCGCATCGCCATCATGCGACGCACGATCTTAGTTGTAACTGGCTCAGATAGGCGCCA   2160
                                     ************************************************************

g4359_CP114861.1_2337142_2337820_    TGTTACCGGCTTTCCACCCACAAGCCCCTGATTGAATTCTATATAAACCCAACTACTTGT   2220
g4654_CP114861.1_7857846_7858524_    TGTTACCGGCTTTCCACCCACAAGCCCCTGATTGAATTCTATATAAACCCAACTACTTGT   2220
                                     ************************************************************

                                                         Tbak-g4359/g4654-R1
g4359_CP114861.1_2337142_2337820_    CTGTTCAAAGCAGGACAGTCTTCTAAACCCTCGTCGCCATTACCAGCTCTTCACTTCAGC   2280
g4654_CP114861.1_7857846_7858524_    CTGTTCAAAGCAGGACAGTCTTCTAAACCCTCGTCGCCATTACCAGCTCTTCACTTCAGC   2280
                                     ************************************************************

g4359_CP114861.1_2337142_2337820_    ATGTCCTATAACAACGACAGTTATGGTTCTTCCTATGACTCTCGGCATGGGTCGTCGAAC   2340
g4654_CP114861.1_7857846_7858524_    ATGTCCTATAACAACGACAGTTATGGTTCTTCCTATGACTCTCGGCATGGGTCGTCGAAC   2340
                                     ************************************************************

g4359_CP114861.1_2337142_2337820_    AATGATAATTACAACTCCAACAATCGCCCTTCTGACAACAATTCCTACGCGTCGTCGAAC   2400
g4654_CP114861.1_7857846_7858524_    AATGATAATTACAACTCCAACAATCGCCCTTCTGACAACAATTCCTACGCGTCGTCGAAC   2400
                                     ************************************************************
```

```
g4359_CP114861.1_2337142_2337820_      AATGTTTCTTACAGTTCTTCCAACAAAGACTCCTCCAACAACCGCTCTTCCGACAATGAC  2460
g4654_CP114861.1_7857846_7858524_      AATGTTTCTTACAGTTCTTCCAACAAAGACTCCTCCAACAACCGCTCTTCCGACAATGAC  2460
                                       ************************************************************

g4359_CP114861.1_2337142_2337820_      ACGTACGGCTCCAACAACCGTTCTTCCAACAATGACTCCTATGGGTCATCCAACAATGAC  2520
g4654_CP114861.1_7857846_7858524_      ACGTACGGCTCCAACAACCGTTCTTCCAACAATGACTCCTATGGGTCATCTAACAATG--  2318
                                       ************************************************** *******


g4359_CP114861.1_2337142_2337820_      TCCTATGGGTCATCCAACAATGATTCCTATGGGTCATCCAACAATGATTCCTATGGCTCC  2580
g4654_CP114861.1_7857846_7858524_      ----------------------ATTCCTATGGGTCATCCAACAATGATTCCTATGGCTCC  2556
                                                             **************************************

g4359_CP114861.1_2337142_2337820_      AACAACCGTTCTTCCAACAATGATTCCTATGGGTCATCCAACAATGATTCCTATGGCTCC  2640
g4654_CP114861.1_7857846_7858524_      AACAACCGTTCTTCCAACAATGATTCCTATGGGTCATCCAACAACGATACGTATGGCTCC  2616
                                       ******************************************** *** * *********

g4359_CP114861.1_2337142_2337820_      AACAACCGTTCTTCCAACAATGATTCCTATGGGTCATCC---------------------  2679
g4654_CP114861.1_7857846_7858524_      AACAACCGTTCTTCCAACAATGACTCCTATGGGTCATCCAACAATGATACGTATGGTTCC  2676
                                       *********************** ***************

g4359_CP114861.1_2337142_2337820_      ---                                                           2679
g4654_CP114861.1_7857846_7858524_      AAC                                                           2679
```

**Supplementary Figure 1**. **Multiple alignment of nucleotide sequences (g4359 and g4654) of 3-Hydroxyathranilate 3,4-dioxygenase (3HAO) from *T. bakamatsutake* genome by ClustalW**.

Capital Letters in yellow indicate CDS. The specific primer sites are shown in bold underlined letter. The primer names are shown upper nucleotide sequence.

**Reference for ClustalW**


Madeira F.; Madhusoodanan N.; Lee J.; Eusebi A.; Niewielska A.; Tivey A.; Lopez R.; Butcher S. The EMBL-EBI Job Dispatcher sequence analysis tools framework in 2024**.** *Nucleic Acids Research*, **2024**, 52, W521–W525. https://doi.org/10.1093/nar/gkae241

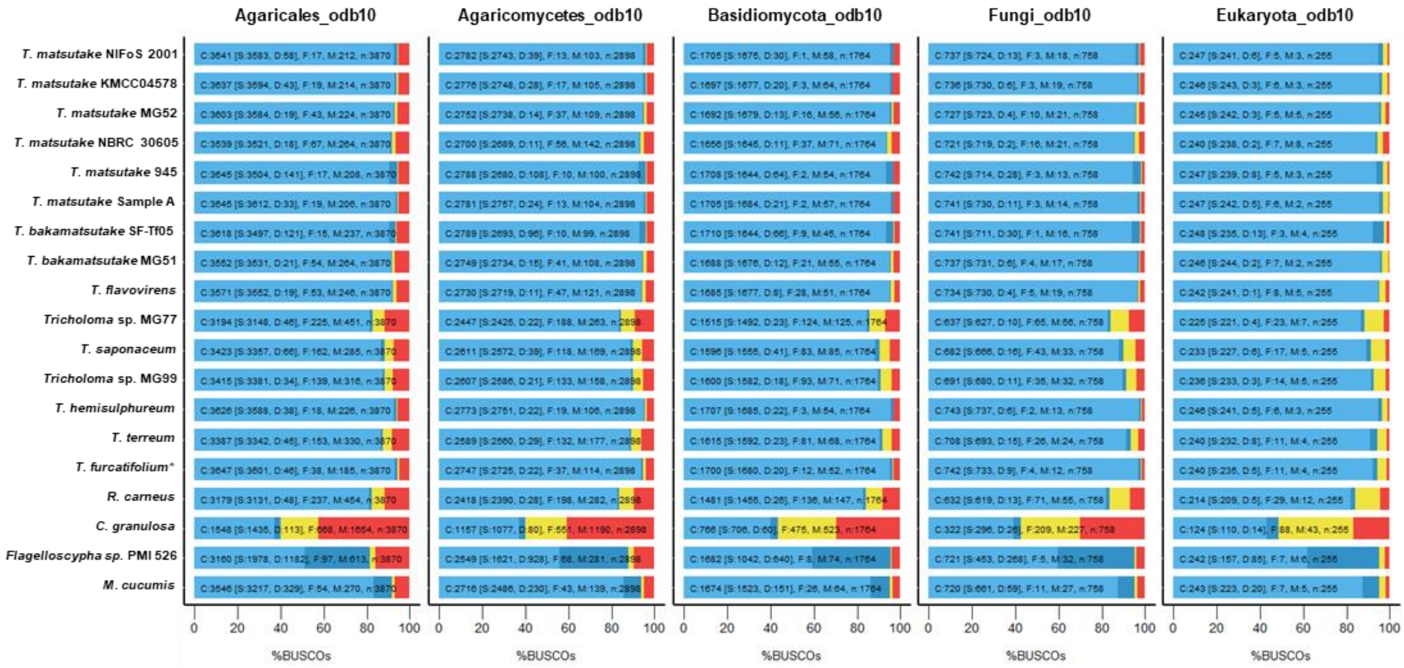


**Supplementary Figure 2. BUSCO conservation across the Tricholomataceae strains.**

The bar graphs display the completeness of BUSCO (Benchmarking Universal Single-Copy Orthologs) profiles for 19 Tricholomataceae strains. The data are organized by five categories of BUSCO datasets: Agaricales_odb10, Agaricomycetes_odb10, Basidiomycota_odb10, Fungi_odb10, and Eukaryota_odb10. Each bar represents the proportion of complete, single-copy BUSCOs (light blue), complete duplicated BUSCOs (dark blue), fragmented BUSCOs (yellow), and missing BUSCOs (red) in the genome assemblies of the different strains.

>JABAJV010008579.1 Tricholoma furcatifolium isolate D33 scaffold_63 4256 4812 –
AACATGATCACTGGTACTTCCCAGGCTGACTGCGCTATCCTCATCATCGCCGGTGGTACTGGTGAGTTCGAGGCTGGTATCT
CCAAGGATGGCCAGACCCGCGAGCACGCTCTCCTTGCCTTCACCCTCGGTGTCAGGCAGCTCATCGTCGCCGTCAACAAG
ATGGACACCACCAAGGTTAGACATCCGAAATCAATGTTACCAACGTGAAAGCTAATATATTGCATTGTAGTGGAGCGAGGACC
GTTTCAATGAAATTATCAAGGAAACTTCTGGCTTCATCAAGAAGGTCGGCTACAACCCCAAGACTGTTGCTTTCGTTCCCATC
TCCGGCTGGCACGGTGACAACATGTTGGAGGAGTCCACCAAGTAAGCATCAATCAAATCAAAAGATCTAACATATACTTACG
CGGTGTTACAGCATGCCCTGGTACAAGGGCTGGACCAAGGAGACCAAGGCTGGTGTCGTCAAGGGCAAGACCCTCCTCG
ATGCCATCGATGCCATCGAGCCCCCCGTCCGTCCTTCCGAGAAGCCTCTCCGTCTTCCTCTCCAGGAT

| Scientific Name | Total Score | Query Cover | E value | Per. Ident | Acc. Len | Accession |
|---|---|---|---|---|---|---|
| Praearthromyces corneri | 1029 | 100% | 0.0 | 100.00% | 557 | OM974133.1 |
| Praearthromyces griseus | 815 | 99% | 0.0 | 93.17% | 556 | OM974132.1 |
| Australocybe olivacea | 654 | 100% | 0.0 | 88.03% | 558 | OM974129.1 |
| Tephrocybe rancida | 617 | 99% | 4e-172 | 86.32% | 559 | OM974135.1 |
| Arthromyces glabriceps | 603 | 99% | 1e-167 | 86.24% | 562 | OM974125.1 |
| Lyophyllum ambustum | 603 | 100% | 1e-167 | 86.42% | 942 | EF421063.1 |
| Cerrena zonata | 599 | 99% | 1e-166 | 86.34% | 891 | OR296401.1 |
| Tephrocybe rancida | 597 | 100% | 5e-166 | 86.17% | 950 | EF421076.1 |
| Tephrocybe rancida | 595 | 99% | 2e-165 | 86.15% | 558 | OM974137.1 |
| Tyromyces sp. X1150 | 592 | 100% | 2e-164 | 85.96% | 1130 | JN710734.1 |

**Supplementary Figure 3. The TEF1A sequences from *Tricholoma furcatifolium* D33 and percent identity matched to the NR database in NCBI.**

Alignment of the *TEF1A* gene sequence from *T. furcatifolium* isolate D33 (scaffold_63: positions 4256–4812) to closely related sequences in the NCBI NR database. The table shows the percent identity, E-value, and query coverage for each match, including *Praearthomyces corneii*, *Praearthomyces griseus*, and *Australocybe rancida* and so on. The sequence alignment highlights strong conservation, with a high percentage of identity, particularly in fungal species closely related to the misidentified *T. furcatifolium*.

>QMFE01004705.1 Tricholoma sp. MG77 scaffold5924_cov41 3464..3810 +
CTTGGTCATTTAGAGGAAGTAAAAGTCGTAACAAGGTTTCTGTAGGTGAACCTGCGGAAGGATCATTATTGAATAAGCTTGAT
TGGGTTGTTGCTGGCTCTTTGGGGCATGTGCACGCCTGACACCAACTTTTCTTACCACCTGTGCACTTTCTGTAGACTTTTG
GGAATACCTCTCGAGGAAACTTGGTTTGAGGATTGCTGTGCGCAAGCAGCCAGCTTTCCTTGCAATTTCCTGTCTATGTTTT
TAATATACCCCTATAGTATGTTACAGAATGTCATTTTAATTGGCTTGATTGCCTTTAAAACCTATACAACTTTCAACAACGGATCT
CTTGGCTCTCGCAT

| Scientific Name | Total Score | Query Cover | E value | Per. Ident | Acc. Len | Accession |
|---|---|---|---|---|---|---|
| Tricholoma sinoportentosum | 582 | 95% | 2e-161 | 98.20% | 712 | NR_185398.1 |
| Tricholoma sinoportentosum | 568 | 95% | 6e-157 | 97.60% | 709 | MF034327.1 |
| Tricholoma sinoportentosum | 566 | 96% | 2e-156 | 97.31% | 708 | MF034248.1 |
| Tricholoma sinoportentosum | 564 | 97% | 7e-156 | 97.03% | 709 | MF034250.1 |
| Tricholoma sinoportentosum | 549 | 90% | 2e-151 | 98.10% | 643 | MW724360.1 |
| Tricholoma sinoportentosum | 549 | 90% | 2e-151 | 98.10% | 643 | MW724359.1 |
| Tricholoma sinoportentosum | 540 | 89% | 1e-148 | 98.06% | 673 | OM867683.1 |
| Tricholoma sejunctum | 525 | 100% | 3e-144 | 94.02% | 862 | AB036899.1 |
| Tricholoma sinoportentosum | 514 | 88% | 7e-141 | 97.07% | 699 | MF034322.1 |
| Tricholoma sinoportentosum | 514 | 88% | 7e-141 | 97.07% | 695 | MF034314.1 |

>QOVE01004689.1 Tricholoma sp. MG99 scaffold6166_cov112 1270..1784 +
CTTGGTCATTTAGAGGAAGTAAAAGTCATAACAAGGTTTCCATAGGTGAACCTGCAGAAGGATCATTATTGAATAAGCTTGGT
TGGGTTGTTGCTGGCTCTTAGGGGCATGTGCACGCCTAACACCAATCTTCTTACCACCTGTGCACCTTTTGTAGACTTGGAT
ATCTCTTGAGGAAACTCGGTATGAGGACTGCTGTGCATCAAAGCCGGCTTTCCTTACATTTCCGGTCTATGTCTTCATATACA
CCATTTGCATGTCTACGAATGTTATTATCGGACTTGACTGTCCAATGAACCTTATACAACTTTCAACAACGGATCTCTTGGCTC
TCGCATCGATGAAGAACGCAGCAAAATGTGATAAGTAATGTGAATTGCAGAATTCAGTGAATCATCGAATCTTTGAACACACC
TTGCGCTCCTTGGTATTCCGAGGAGCATGCCTGTTTGAGTGTCATGAAATTCTCAACCTTTTCAGCTTTTCTGAAGTTGATCA
GGCTTGGATGTGGGAGT

| Scientific Name | Total Score | Query Cover | E value | Per. Ident | Acc. Len | Accession |
|---|---|---|---|---|---|---|
| Tricholoma albobrunneum | 883 | 98% | 0.0 | 98.22% | 714 | MF034232.1 |
| uncultured Tricholoma | 880 | 100% | 0.0 | 97.48% | 810 | EU046039.1 |
| Tricholoma ustale | 880 | 99% | 0.0 | 97.66% | 686 | AF458437.1 |
| Tricholoma ustale | 880 | 99% | 0.0 | 97.66% | 685 | AF458436.1 |
| Tricholoma ustale | 876 | 100% | 0.0 | 97.29% | 739 | LC574900.1 |
| Tricholoma albobrunneum | 876 | 100% | 0.0 | 97.29% | 735 | LC574893.1 |
| Tricholoma ustale | 876 | 100% | 0.0 | 97.29% | 735 | LC574884.1 |
| Tricholoma ustale | 876 | 100% | 0.0 | 97.29% | 874 | AB036894.1 |
| Tricholoma albobrunneum | 874 | 97% | 0.0 | 98.20% | 703 | MF034254.1 |
| Tricholoma ustale | 872 | 100% | 0.0 | 97.10% | 734 | LC574897.1 |

**Supplementary Figure 4. The ITS sequences from *Tricholoma* spp. MG77 and MG99 and percent identity matched to the ITS database in NCBI.**

The figure illustrates the alignment of ITS sequences from *Tricholoma* spp. MG77 and MG99 with the ITS database in NCBI, highlighting their percent identity matches. The top panel shows the ITS sequence from *Tricholoma* sp. MG77 (scaffold5924_cov41, positions 3464-3810), which displays high similarity to *Tricholoma sinopotentinum* strains, with percentage identity 98.20% and strong E-value. The bottom panel presents the ITS sequence from *Tricholoma* sp. MG99 (scaffold6166_cov112, positions 1270-1784), revealing close similarity primarily to *Tricholoma albobrunneum*, with percentage identity 98.22% and significant E-value. The accompanying tables summarize the match statistics, including total score, query coverage, E-value, percent identity, alignment length (Acc. Len.), and the NCBI accession numbers for each match.

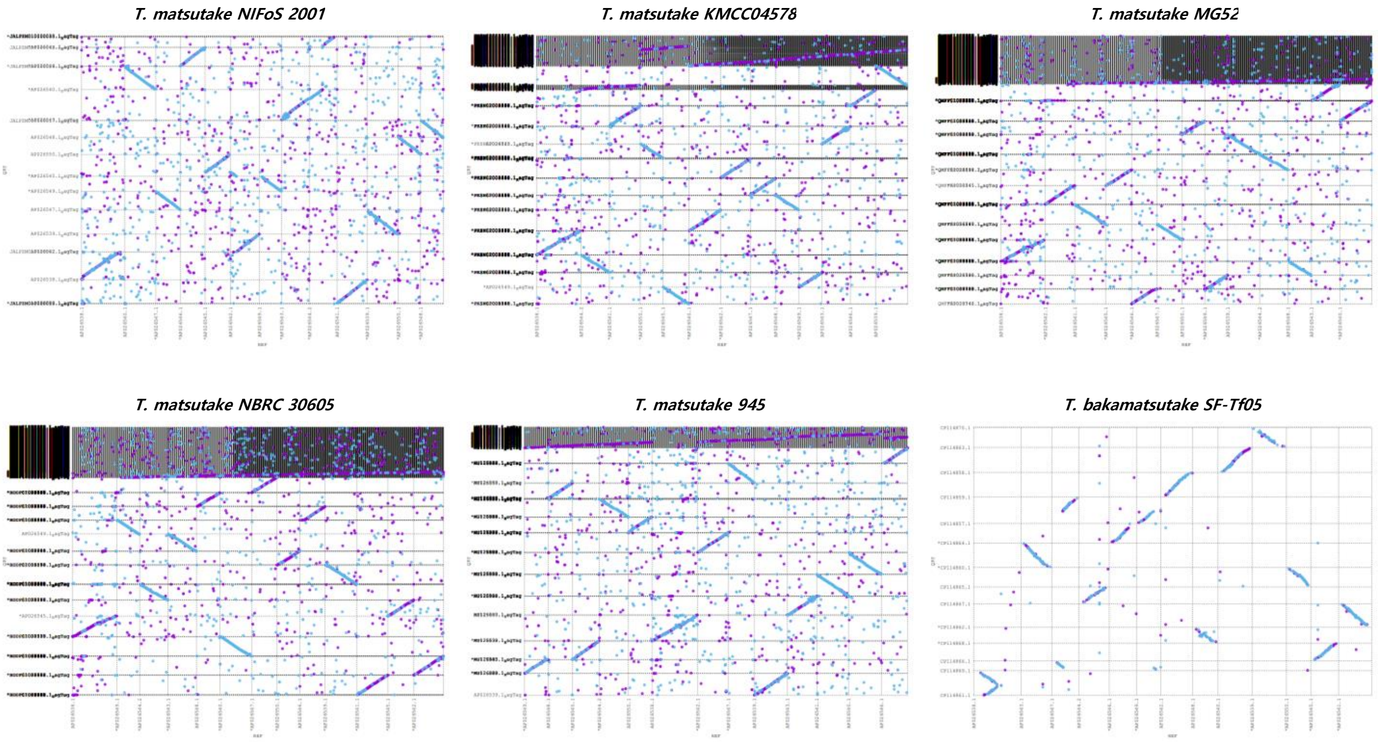


**Supplementary Figure 5. Synteny relationships of *T. matsutake* strains against the reference strain Sample A.**

The figure displays synteny dot plots comparing various *T. matsutake* strains against the reference strain, Sample A, to assess the conservation and rearrangement of genomic regions across the strains. Each panel represents a different strain, where dots indicate homologous regions between the genomes, with diagonal lines signifying conserved synteny blocks. Disruptions in these lines or scattered dots suggest genomic rearrangements, inversions, or other structural variations relative to the reference.

*T. matsutake* *T. bakamatsutake*

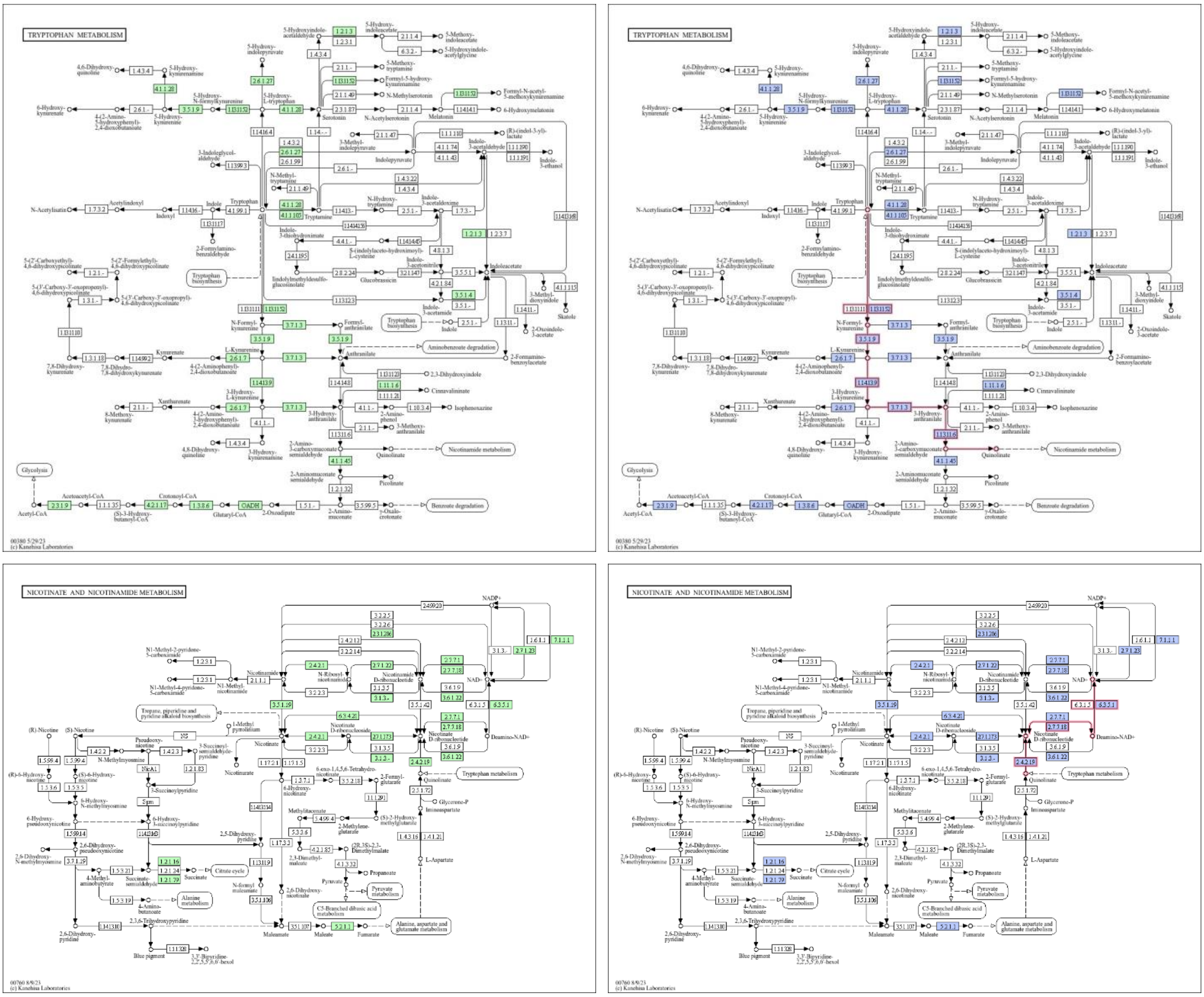


**Supplementary Figure 6. Comparative analysis of tryptophan, nicotinate, and nicotinamide metabolism pathways in *T. matsutake* and *T. bakamatsutake*.**

The KEGG pathway maps compare tryptophan metabolism (top) and nicotinate and nicotinamide metabolism (bottom) between *T. matsutake* (left) and *T. bakamatsutake* (right). Genes identified in all *T. matsutake* strains are highlighted in green, while those in all *T. bakamatsutake* strains are highlighted in blue. Red highlights indicate the presence of a complete pathway within each species.

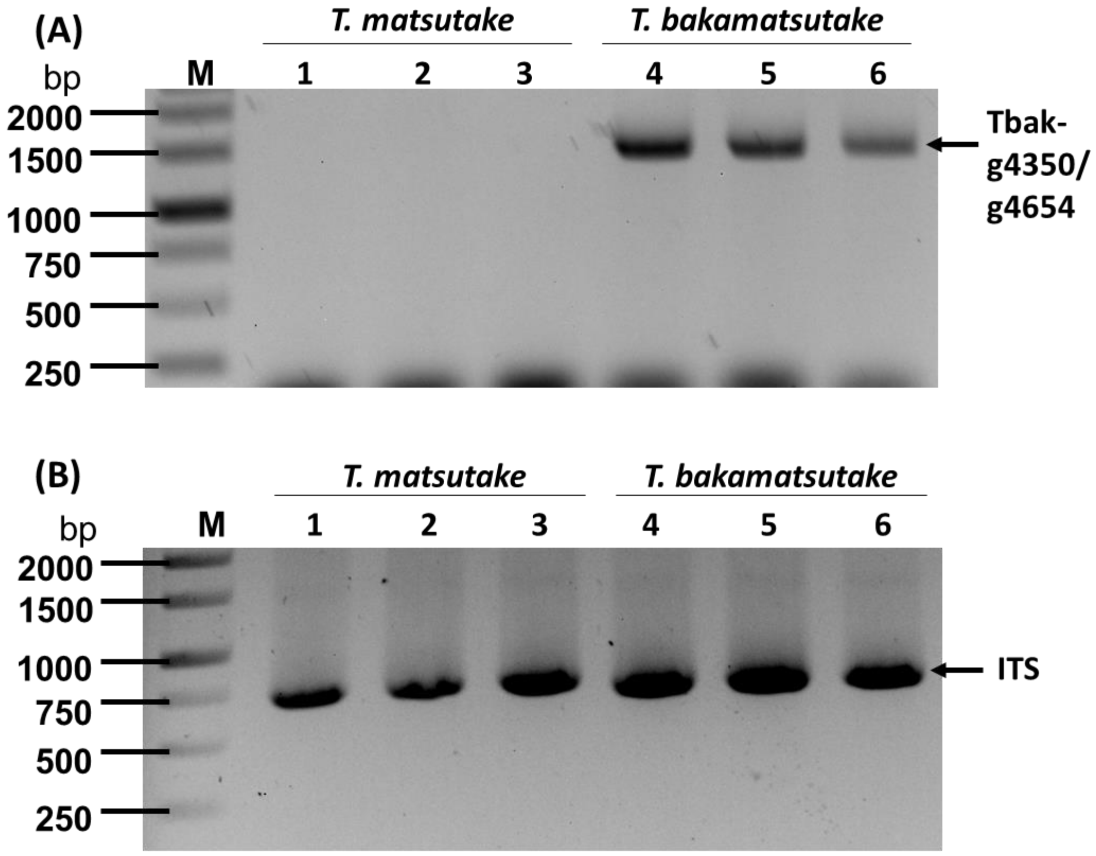


**Supplementary Figure 7. Specific identification of 3-hydroxyanthranilate 3.4-dioxygenase (*3HAO*) gene from genomic DNA of *T. matsutake* and *T. bakamatsutake* by PCR amplification.**

(A) 3HAO and (B) Internal control in *T. matsutake* and *T. bakamatsutake*. M: GeneRuler Ladder, Lane 1~3: *T. matsutake* strain NIFoS 434, 561 and 2001, respectively. Lane 4~6: *T. bakamatsutake* strain NIFoS 3833, 4757 and 4982, respectively.
